# Evaluation of the Inhibition of Carbohydrate Hydrolyzing Enzymes, the Antioxidant Activity, and the Polyphenolic Content of *Citrus limetta* Peel Extract

**DOI:** 10.1155/2014/121760

**Published:** 2014-12-21

**Authors:** Eduardo Padilla-Camberos, Estefania Lazcano-Díaz, José Miguel Flores-Fernandez, Moses S. Owolabi, Kirk Allen, Socorro Villanueva-Rodríguez

**Affiliations:** ^1^Centro de Investigación y Asistencia en Tecnología y Diseño del Estado de Jalisco, A.C., Avenida Normalistas 800, Colinas de la Normal, 44270 Guadalajara, JAL, Mexico; ^2^Department of Chemistry, Lagos State University, PMB 0001, Lasu Post Office, Ojo, Lagos, Nigeria; ^3^Lancaster Medical School, Lancaster University, Lancaster LA1 4YG, UK

## Abstract

Type 2 diabetes mellitus is one of the most frequent causes of death in Mexico, characterized by chronic hyperglycemia. One alternative strategy for this metabolic abnormality is inhibiting the enzymes responsible for the metabolism of carbohydrates. We evaluated whether the aqueous *Citrus limetta* peel extract could inhibit the metabolism of carbohydrates. We found that this extract inhibited primarily the enzyme *α*-amylase by 49.6% at a concentration of 20 mg/mL and to a lesser extent the enzyme *α*-glucosidase with an inhibition of 28.2% at the same concentration. This inhibition is likely due to the high polyphenol content in the *Citrus limetta* peel (19.1 mg GAE/g). Antioxidant activity of the *Citrus limetta* peel demonstrated dose-dependent antioxidant activity, varying from 6.5% at 1.125 mg/mL to 42.5% at 20 mg/mL. The study of these polyphenolic compounds having both antihyperglycemic and antioxidant activities may provide a new approach to the management of type 2 diabetes mellitus.

## 1. Introduction

Diabetes mellitus (DM) is a metabolic disorder, characterized by hyperglycemia and by disturbances in the metabolism of fat and protein, resulting from defects in insulin secretion and/or insulin action [1]. Type 2 DM or noninsulin dependent DM is the most frequent form with 90–95% of DM cases [2]. Currently, Mexico ranks ninth in prevalence of type 2 DM [3, 4]. Several studies have been conducted to elucidate the possible biochemical mechanisms involved in the pathogenesis of type 2 DM, but the exact mechanism is still unclear. Hyperglycemia, one of the main characteristics of type 2 DM, is considered the main cause of complications from diabetes [5]. Several pharmacological approaches have been used to improve diabetes treatment through different modes of action including stimulating insulin release, inhibiting gluconeogenesis, increasing the number of glucose transporters, and reducing glucose absorption from the intestine [2], which is achieved with enzyme inhibitors such as acarbose, voglibose, and miglitol [6]. Gastrointestinal side effects make pharmacological approaches less attractive as therapeutic agents, which makes natural remedies viable alternatives [7]. Previous studies have shown that the ability to inhibit enzymes is responsible for carbohydrate metabolism by different natural compounds [5]. Over 200 pure bioactive compounds isolated from plants have shown the effect of reducing blood glucose [8], several of which are polyphenolic compounds [9].* Citrus limetta*, commonly known as sweet lime, is an edible fruit from Central America [10]. It is small, between oval and spherical, with a greenish yellow peel, rich in polyphenols, flavonoids, flavanones, and flavones [11]. This fruit is typically used for human consumption, and it has also been used for cholesterol control and inflammation regulation as well as digestive disorders and as a modulator of blood pressure [12].* Citrus limetta* is comprised of 8–10% peel, which generally is a byproduct without any use, becoming an environmental problem [13]. Recent studies have shown interest in the possible beneficial effects of foods rich in polyphenols [14], which have different activities, among the most interesting of which are carbohydrate metabolism by inhibiting the *α*-glucosidase and *α*-amylase enzymes responsible for carbohydrate digestion [15], avoiding the chronic hyperglycemia symptoms that characterize type 2 DM.

## 2. Materials and Methods

### 2.1. Plant Material

*Citrus limetta* was purchased in Jalisco, Mexico. The peel was dried at 37°C, then ground and sieved (300 *μ*m), and packaged until use. *Citrus limetta *peel extract. 0.5 g sample was homogenized in 25 mL of distilled water and then filtered on Whatman 40 to remove particles. The obtained sample was placed in an amber bottle and kept at 4°C until use.

### 2.2. Total Polyphenolic Assay

Total phenolic content was determined using Folin-Ciocalteu method [16]. 15 *μ*L of extract sample at different concentrations (20, 10, 5, 2.5, and 1.125 mg/mL) was mixed with 750 *μ*L of 0.2 M Folin-Ciocalteu reagent and 600 *μ*L of 7.5% sodium carbonate. The absorbance was measured at 765 nm after incubation in the dark for 2 hours. Gallic acid was used as standard. The total polyphenol content was expressed as milligrams of gallic acid equivalents (GAE) per gram of the sample.

### 2.3. Antioxidant Activity

Antioxidant activity was evaluated by the 2,2-diphenyl-2-picrylhydrazyl (DPPH) method [17]. 20 *μ*L of the extract was mixed at different concentrations (20, 10, 5, 2.5, and 1.125 mg/mL), with 200 *μ*L of 36 *μ*M DPPH in 80% methanol and then was incubated in the dark for 30 min. The absorbance was measured at 515 nm. Ascorbic acid at 300 *μ*g/mL was used as standard. The total antioxidant activity (%TAA) was expressed as the percentage inhibition of DPPH radical and determined with the following equation:

(1) $$\begin{array}{c} \%\text{TAA}=\frac{\text{Abs}\;\;\text{control}-\text{Abs}\;\;\text{sample}}{\text{Abs}\;\;\text{control}}\times 100, \end{array}$$

where %TAA is antioxidant activity and Abs is absorbance.

### 2.4. *α*-Glucosidase Inhibition Essay

Inhibition of *α*-glucosidase enzyme was determined colorimetrically by monitoring the release of 4-nitrophenol from 4-nitrophenyl-*α*-D-glucopyranoside (pNPG) [18]. 50 *μ*L of extract at different concentrations (20, 10, 5, 2.5, and 1.125 mg/mL) was preincubated with 100 *μ*L of *α*-glucosidase enzyme (1 U/mL) (Sigma Aldrich). The reaction was carried out by adding 50 *μ*L pNPG followed by 30 minutes incubation at 25°C. Acarbose was used as standard. Phosphate buffer was used as blank. The inhibition percentage was determined with the following equation:

(2) %Inhibition=100×Abs Cx−Abs Co−Abs Mx−Abs MoAbs Cx−Abs Co,

where Abs *C*_*x*_ is absorbance of control at minute 5, Abs *C*_*o*_ is absorbance of control at minute 0, Abs *M*_*x*_ is absorbance of sample at minute 5, and Abs *M*_*o*_ is absorbance of sample at minute 0.

### 2.5. *α*-Amylase Inhibition Essay

250 *μ*L of sample at different concentrations (20, 10, 5, 2.5, and 1.125 mg/mL) was preincubated with 250 *μ*L of *α*-amylase enzyme (2 U/mL) (Sigma Aldrich); then 250 *μ*L of 1% starch was added and the sample was incubated at 25°C for 10 minutes. The reaction was stopped by adding 500 *μ*L 3,5-Dinitrosalicylic acid (DNS). The sample was immediately incubated in a water bath at 95°C for 5 minutes and then was cooled to room temperature and 5 mL of distilled water was added. Acarbose was used as standard and phosphate buffer as blank. The absorbance was measured at 540 nm. The inhibition percentage was determined with the following equation:

(3) $$\begin{array}{c} \%\text{TAA}=\frac{\text{Abs}\;\text{control}-\text{Abs}\;\text{sample}}{\text{Abs}\;\text{control}}\times 100, \end{array}$$

where Abs is the absorbance.

### 2.6. Statistical Analysis

All experiments were executed in triplicate and the data were expressed as mean ± standard deviation. Statistical comparisons were performed by one-way analysis of variance followed by Least Significant Difference (*P* < 0.05) using Statgraphics XVI software.

## 3. Results

### 3.1. Total Polyphenolic Content

The* Citrus limetta* extract showed a total phenolic content of 19.1 ± 1.6 mg GAE/g in a sample concentration 20 mg/mL.

### 3.2. Antioxidant Activity

**Figure 1:**
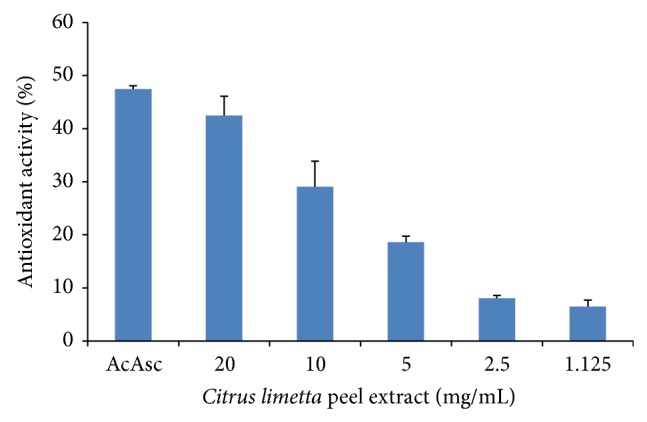
Antioxidant activity at different concentrations of* Citrus limetta* peel extract. Data mean ± S.D. are plotted (*n* = 3). Ascorbic acid (AcAsc) at 300 *μ*g/mL was used as standard.

The* Citrus limetta* extract showed high antioxidant activity and was dose-dependent (Figure 1). The activity was 42.5, 29.1, 18.6, 8.1, and 6.5% at concentrations of 20, 10, 5, 2.5, and 1.125 mg/mL, respectively (Figure 1).

### 3.3. *α*-Glucosidase Inhibition

**Figure 2:**
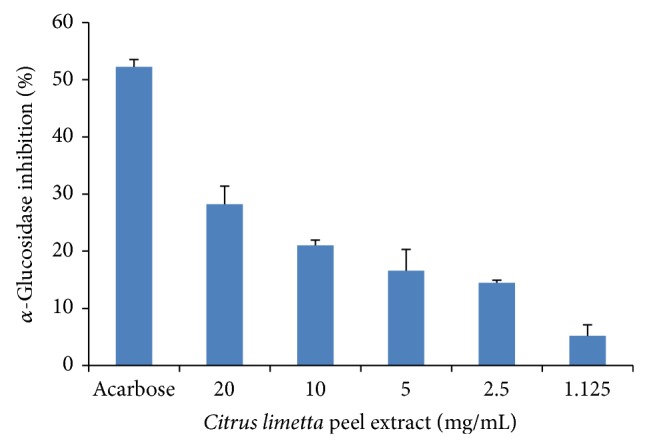
*α*-Glucosidase inhibition at different concentrations of* Citrus limetta* peel extract. Data mean ± S.D. are plotted (*n* = 3). Acarbose was used as standard.

The enzymatic activity of *α*-glucosidase was inhibited by all concentrations of the* Citrus limetta* peel extract. The inhibition was in a dose-dependent manner. The activity was 28.2, 21.0, 16.6, 14.5, and 5.2% at concentrations of 20, 10, 5, 2.5, and 1.125 mg/mL, respectively (Figure 2).

### 3.4. *α*-Amylase Inhibition

**Figure 3:**
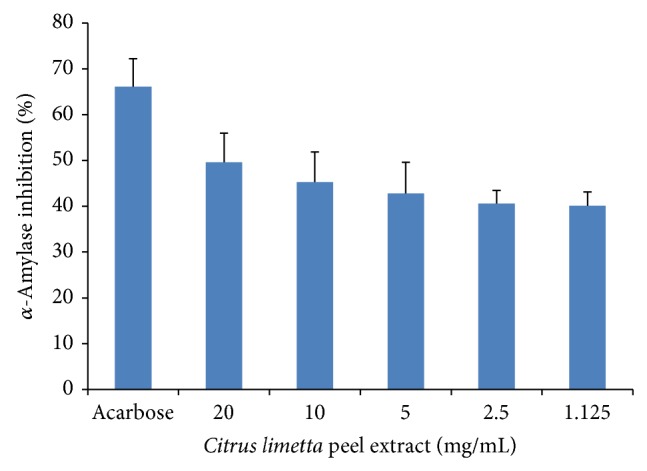
*α*-Amylase inhibition at different concentrations of* Citrus limetta* peel extract. Data mean ± S.D. are plotted (*n* = 3).

The enzymatic activity of *α*-amylase was inhibited by the* Citrus limetta* extract by 49.6, 45.3, 42.8, 40.6, and 40.1% at concentrations of 20, 10, 5, 2.5, and 1.125, respectively (Figure 3).

## 4. Discussion

Hyperglycemia is a metabolic abnormality common in the people with type 2 DM. Hyperglycemia is characterized by increased levels of glucose in the blood; thus it is essential to maintain the glucose levels close to normal [19]. The enzyme *α*-amylase is an endoglucanase that hydrolyzes polysaccharides, and *α*-glucosidase is located in the membrane of the surface of the edge in brush of intestinal cells [20, 21]. These enzymes are key for digestion and carbohydrates absorption, thus regulating the glucose levels in blood [2].

In this study, we evaluated the ability of* Citrus limetta* peel extract to inhibit the enzymatic activity of *α*-glucosidase and *α*-amylase and observed a positive correlation between the total content of polyphenols and enzymatic activity. The dose-dependent relationships suggest that polyphenols of the* Citrus limetta* extract are partly responsible for the inhibition of the enzymatic activity.

Some studies have shown the power with which polyphenols inhibit activities of *α*-glucosidase and *α*-amylase [22, 23]. Flavonoids weakly inhibit *α*-glucosidase, though they are often potent inhibitors of *α*-amylase [22]. This coincides with our obtained results because* Citrus limetta* peel extract was a weak inhibitor of *α*-glucosidase activity but a potent inhibitor of the enzymatic activity of *α*-amylase. This difference could be due to different polyphenols present in the extract.

The polyphenolic compounds may also be indirectly beneficial to DM by the chelating metallic ions [24] or by activating the antioxidant enzyme expression [25]. A high total content of polyphenols is not always translated to a high antioxidant activity. The antioxidant activity appears depending on the position and degree of hydroxylation and conjugation [26].

It has been found that peels are the main sources of polyphenols from fruits [27], since these participate in purification of free radicals and may be involved in the inhibition of enzymatic activity [16].

According to the results obtained at different concentrations, we observed that the antioxidant and inhibitory activity of the enzymes which hydrolyze carbohydrates depends on the concentration of polyphenols present in the* Citrus limetta* peel extract.

## 5. Conclusion

The aqueous extract of* Citrus limetta* peel showed a potent inhibition of the enzymatic activity of *α*-glucosidase and *α*-amylase, which could be related to the polyphenol content. However, further studies are required using polyphenolic isolated compounds, as well as studies* in vivo*. The results give the possibility that the peel of* Citrus limetta*, which is generally discarded, becoming an ecological problem, could be exploited for use as an alternative for the control of hyperglycemia in people with type 2 DM.
